# Spatial light modulation for interferometric scattering microscopy

**DOI:** 10.1111/jmi.13347

**Published:** 2024-08-26

**Authors:** Vivien Walter, Christopher Parperis, Yujie Guo, Mark Ian Wallace

**Affiliations:** ^1^ Department of Chemistry King's College London, Britannia House London UK

**Keywords:** interferometric scattering microscopy, PSF engineering, spatial light modulation

## Abstract

Interferometric scattering (iSCAT) microscopy enables high‐speed and label‐free detection of individual molecules and small nanoparticles. Here we apply point spread function engineering to provide adaptive control of iSCAT images using spatial light modulation. With this approach, we demonstrate improved dynamic spatial filtering, real‐time background subtraction, focus control, and signal modulation based on sample orientation.

## LAY DESCRIPTION

In this paper, we apply spatial light modulation to manipulate the illumination properties of an interferometric scattering microscopy. This technique, called iSCAT, can image individual molecules and nanoparticles without the need for fluorescent labels. We added a digital display device that can modulate the pattern and phase of the laser illumination. This allows us to control the light used to make images. By changing how we use the light, we can optimise the contrast of our images, make rapid changes in focus without mechanical movement, and explore the alignment of gold nano rods.

## 1 INTRODUCTION

Interferometric scattering microscopy (iSCAT) is a sensitive label‐free optical technique for imaging nanoscopic objects, including individual biomolecules.^1^, ^2^, ^3^ Notably it is a promising method with which to tackle some of the limitations of single‐molecule fluorescence (SMF) microscopy. Indeed, while SMF methods have undoubtedly revolutionised our understanding of biology,^4^ and while advances in spatial and temporal resolution continue apace,^5^, ^6^, ^7^ the use of fluorescence as an image contrast mechanism presents some inherent experimental restrictions. For example, fluorescent labelling can alter biomolecule properties,^8^, ^9^ photobleaching prevents imaging for extended periods of time,^10^ and optical saturation provides a hard limit to the sampling rate at which a fluorescence process can be imaged.^11^ Due to its nature, iSCAT circumvents the need for fluorescence labelling, relying instead on the interference between a local reference light field and the elastic scattering from an individual object.^11^ Although typically weaker, this signal is not subject to the same limitations and is dependent on wavelength, particle and medium permittivity, and volume.

Albeit in a shot‐noise‐limited measurement the SNR is independent of the contrast, small particle volumes understandably produce weak signals, and so image filtering is desirable to improve detection of small nanoparticles or single protein molecules. For example, control of the relative magnitude of scattered and reference signals has been reported as a mechanism to enhance the overall sensitivity of interferometric microscopy: Image contrast has been boosted by use of a half‐silvered mirror to selectively reduce background signal in the output light path,^12^ and spatial filtering has been exploited, both by use of diaphragms^13^ and partially reflective metallic masks^14^ to selectively attenuate spatial frequencies in the image, and hence control the overall detected contrast. A limitation to date is the fixed nature of the signal modulation these methods provide, with filtering engineered specifically for a given experiment. Spatial light modulators (SLMs) are a well‐established technology to provide dynamic, real‐time control of the light field, widely applicable in microscopy.^15^ For example, SLM‐based adaptive optics can be used for aberration correction^16^, ^17^, ^18^, ^19^ and to achieve subdiffraction‐limited information, either by generating structured illumination fields^20^, ^21^ or by re‐engineering the point spread function.^22^ Similarly SLMs can be used as holographic lenses, to select focal planes and provide depth information.^23^, ^24^, ^25^, ^26^, ^27^

Specifically, we sought to quantify the potential of this method to provide adaptable, real‐time control of contrast enhancement, focus, background subtraction and polarisation through imaging samples of gold nanoparticles (AuNPs) and gold nanorods (AuNRs).

## 2 RESULTS

### 2.1 Instrument construction

**FIGURE 1:**
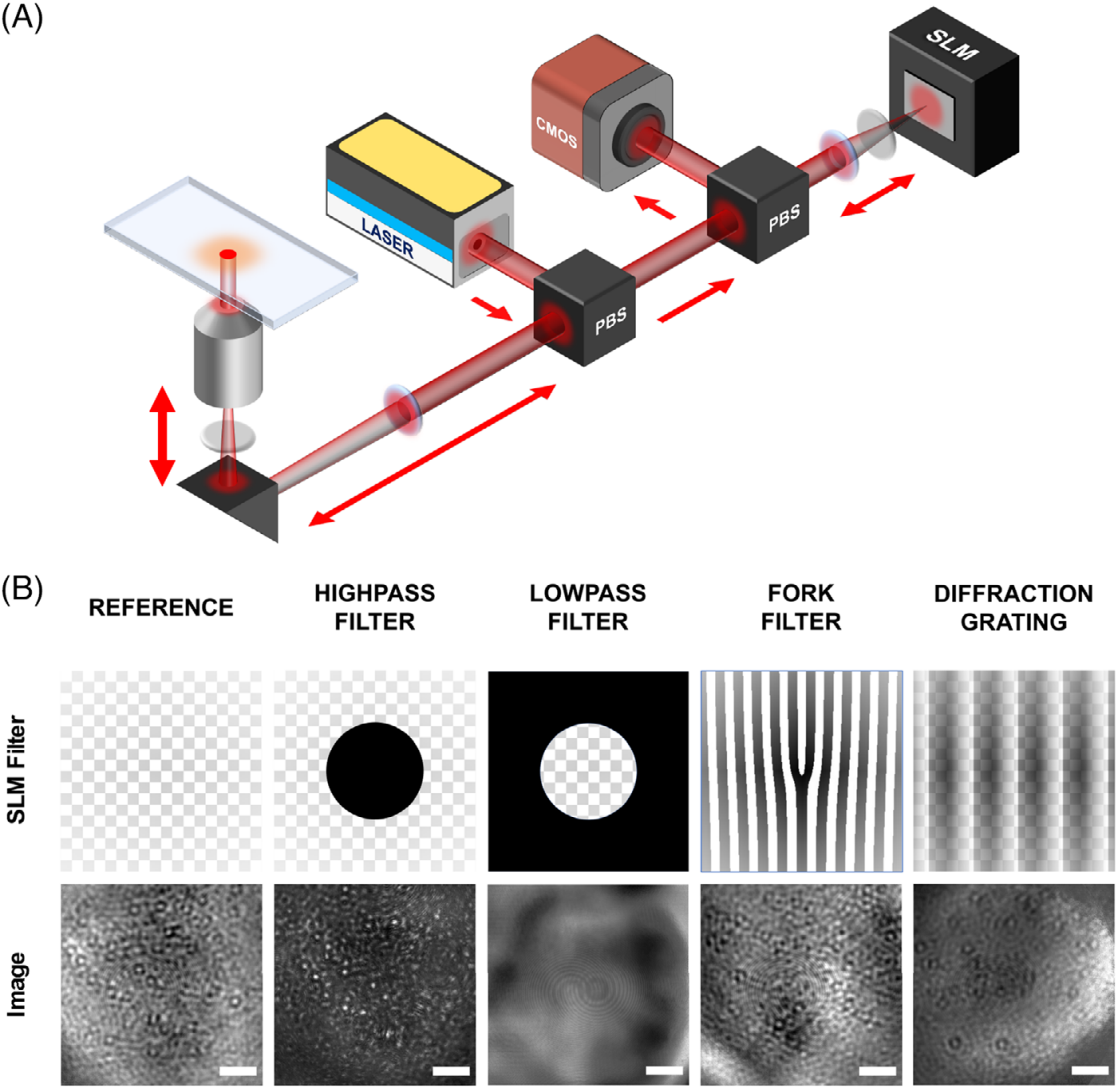
(A) Simplified illustration of the iSCAT optical setup including the spatial light modulator (SLM) in the light path. Blue disks represent the main focussing/collimating lenses, while grey disks represent quarter wave plates. (B) A selection of different filter types that the SLM can project into frequency space. Top row: illustrations of filters as they are displayed on the SLM. Bottom row: iSCAT response to each filter for samples of AuNPs (40 nm). Scale bars 4 μm.

A simplified illustration of the optical setup is shown in Figure 1A. Briefly, a a single‐mode diode laser (640 nm, iBeam smart PT, Toptica, Munich GE) was directed into a microscope objective (Plan Apo 100×/1.40 Oil ∞/0.17 WD 0.13 DIC N2, Nikon) using a polarising beam splitter (PBS) and quarter‐wave plate to isolate back scattering from the image and provide epi‐illumination of the sample. A second PBS and quarter‐wave plate was then used to project a conjugate back‐focal plane onto a reflective SLM (920×1152 pixel, Meadowlark Optics, Frederick, CO, USA). Finally reflection from the SLM is returned through the second PBS and quarter wave plate to produce a focused image on a CMOS camera (MV1‐D1024E‐160‐CL, Photonfocus, Lachen CH). XYZ position of the sample was controlled using a piezoelectric stage (P‐545‐3R8S, Physik Instrumente, Karlsruhe, GE). Other optomechanical components were either purchased from Thorlabs (Newton, NJ, USA) or custom designed and fabricated.

The use of a SLM enables selective retardation of the optical wavefront at particular x,y positions, corresponding to each pixel of the device. When placed at a conjugate back focal plane, the SLM acts to induce a phase delay in specific spatial frequencies, which are subsequently occluded by use of a quarter‐wave plate and PBS. Patterns displayed on the SLM are filters which can be classified depending on the effect they have on the propagating wavefront. Figure 1B illustrates a range of potential applications for wavefront control. Filters include, but are not limited to, high‐, low‐ and band‐pass filters, which selectively suppress ranges of spatial frequencies above or below a predetermined threshold^28^; fork filters, which may control the optical angular momentum of optical vortices^29^; and diffraction gratings, which can induce a lateral shift to duplicate an image.^30^

With our instrument established, we sought to quantify the effects of three specific filter types of interest in iSCAT microscopy: (1) background‐reduction via high‐pass filtering; (2) focus‐control via Fresnel filters; and (3) orientation determination via directional filtering.

### 2.2 Contrast optimisation

Interference contrast can be optimised by controlling the relative magnitude of reference (background) intensity to that from a scattering object of interest.^12^ High‐pass filtering is a simple spatial filter that removes a significant portion of the background intensity, along with typically unwanted low‐frequency information while retaining the high‐frequency signal of interest. By controlling the frequencies cut by the filter, the effective contrast can be controlled.

**FIGURE 2:**
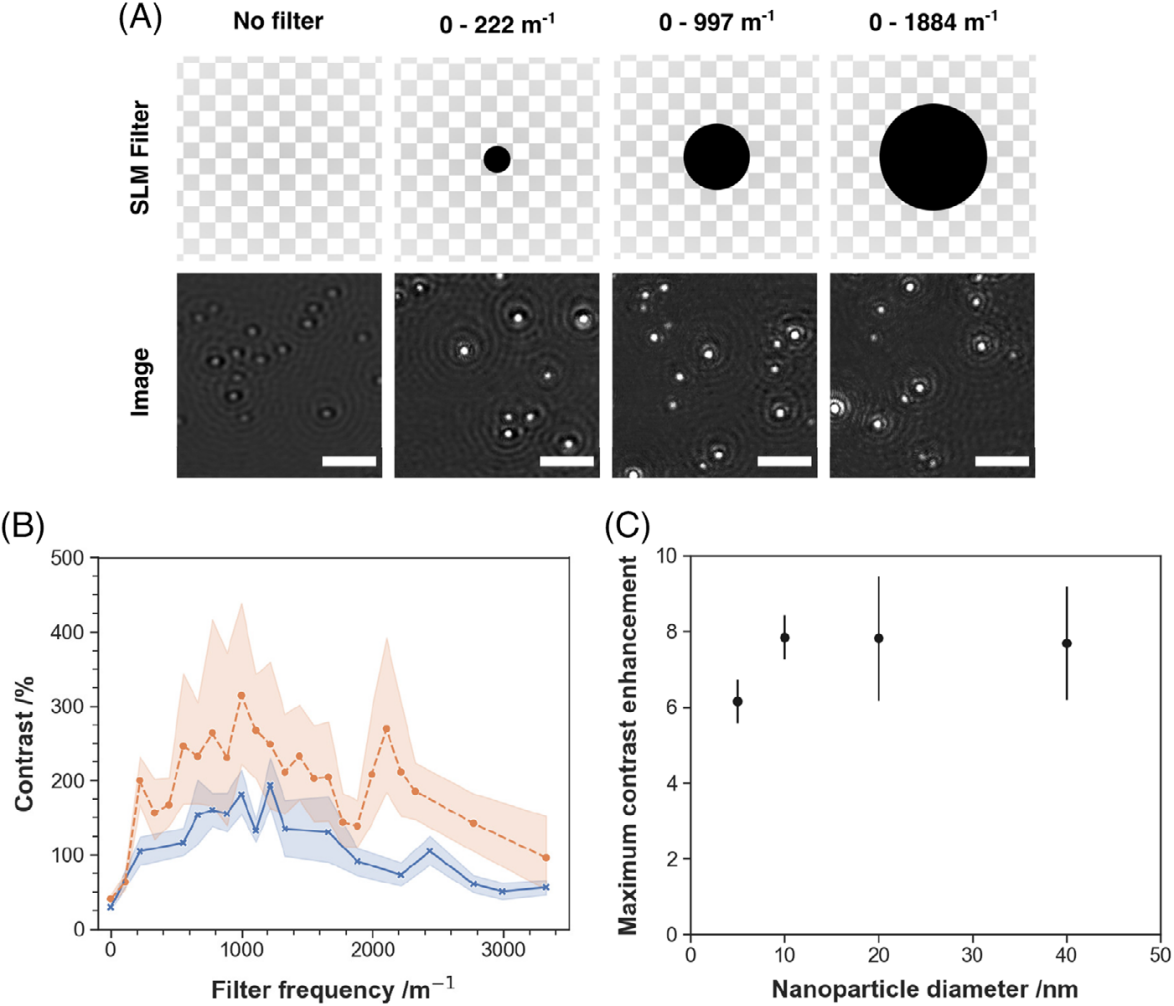
(A) Effect of high‐pass filtering on a sample of 5 nm AuNPs. Illustration of the corresponding filters are shown on the top row. Scale bars 3 μm. (B) Evolution of interference contrast with spatial frequency measured for two sizes of AuNPs: 5 (blue) and 40 (red) nm. Markers represent the average contrast while the areas represent the respective 95% confidence interval. (C) Contrast enhancement factors for various AuNPs sizes at the experimentally determined optimal high‐pass frequency cutoff of 997 $m^{-1}$.

A sample of 5 nm gold particles was imaged on our iSCAT microscope. Different high‐pass filters, consisting of black circles of varying diameter, were projected by the SLM and the subsequent images were recorded. Examples are shown in Figure 2A. The pixel diameters of each filter are reported as the corresponding occluded spatial frequencies.

Figure 2 shows the measured dependence of nanoparticle contrast with SLM high‐pass filter frequency. These data were collected from ∼100 nanoparticles over 3 samples. Nanoparticles were segmented using the TrackPy python module^31^ with minimal intervention – simply analysing top 64% of pixel intensities following noise and background preprocessing. Here, contrast was determined by the division of the raw image by the background image following lateral displacement and median averaging.^32^ Removing spatial frequencies below 997 ± 55 $m^{-1}$ provided the greatest increase in contrast (Figure 2B). The same optimal effective frequency cutoff was found for all AuNPs samples (5, 10, 20 and 40 nm) using the same protocol (Figure 2C), providing a mean increase in contrast by a factor of 7 ± 4. The independence of optimal high‐pass cutoff frequency and AuNPs size is expected; as all particles are significantly below the diffraction limit, only the absolute contrast changes with nanoparticle size. Correspondingly, the factor by which each filter increases contrast is observed to be independent of object size. The variation of contrast with filter frequency is not a smooth function; we interpret the local variations in contrast with spatial frequency as due to the digital nature of the SLM spatial response across pixels.

To picture the effects of this spatial filter, consider the optimal cutoff frequency expressed as a spatial period; any feature of the image larger than 0.67 ± 0.04 μm will be filtered from the image. This optimal spatial extent is, unsurprisingly, similar to the width of the point spread function of our imaging system (σ≈0.61±0.04μm, Figure S1).

### 2.3 Focus control

**FIGURE 3:**
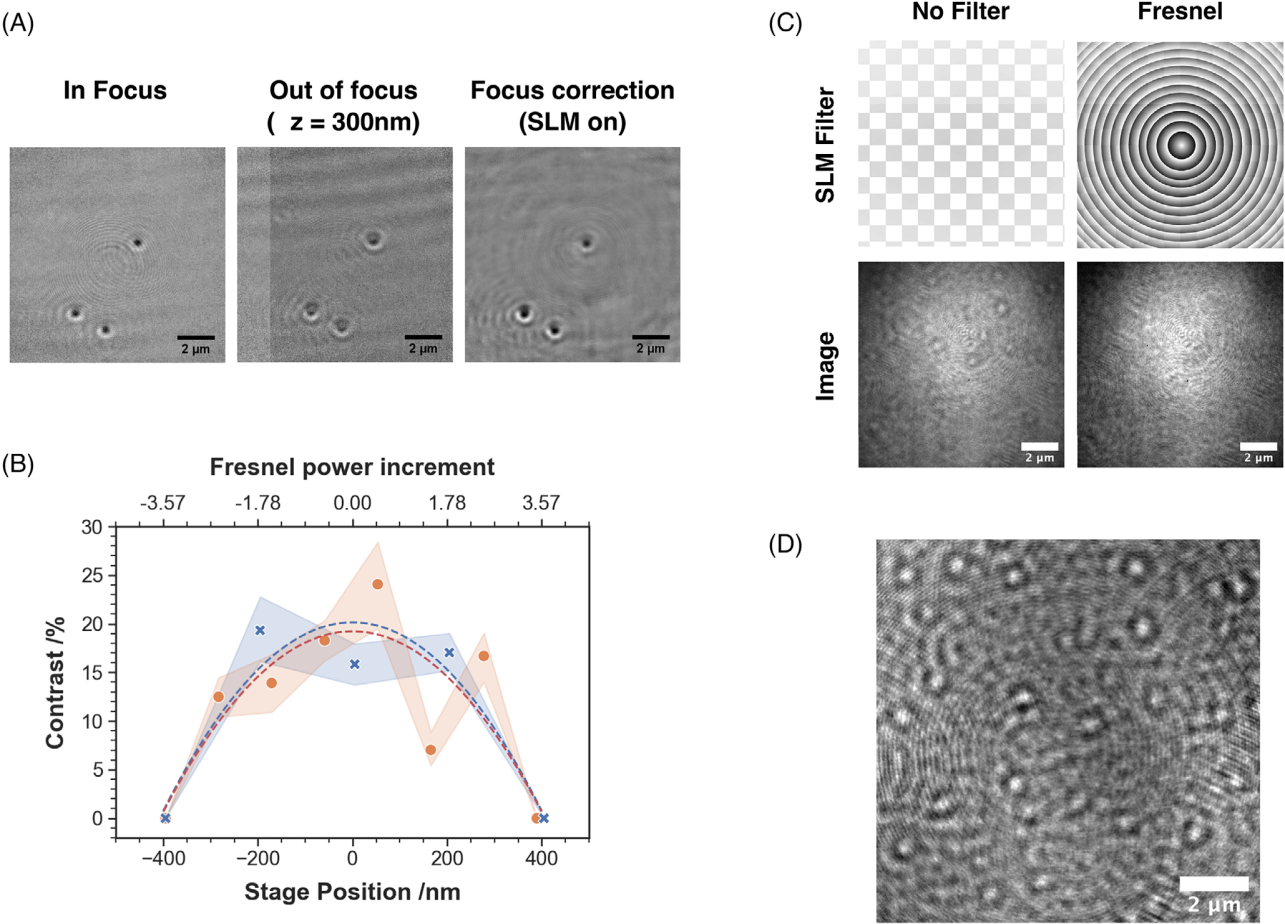
(A) Sample of 40 nm AuNPs, first brought into focus (left) and then drifted out of focus by 300 nm (centre) using the microscope stage. The particles are brought back into focus (right) using a Fresnel pattern and no movement of the stage. (B) Evolution of image contrast for 40 nm gold particles using different Fresnel patterns at fixed stage position (red) or different stage positions with no Fresnel pattern applied (blue). Markers represent the average contrast while the areas represent the respective CI 95 of the markers determined for 40 nanoparticles. (C) Unprocessed images of a AuNPs sample entirely defocused upon projection of a the Fresnel pattern. (D) Result of subtracting the defocused image from the in‐focus image. We propose this strategy as a method for real‐time background correction, equally effective for imaging either diffusing or static objects.

Encoding Fresnel patterns on the SLM converts the device into a diffraction‐based lens, enabling the SLM to rapidly select the focal plane which forms an image on the CMOS detector. Thus for a fixed distance between the objective and the sample, modification of Fresnel pattern properties in turn modifies the position of the object plane, emulating axial movement of the objective lens relative to the sample. A representative example of use of the SLM for focus adjustment is presented in Figure 3A. 40 nm AuNPs bound to a microscope coverslip were brought into focus using the piezoelectric stage. Subsequently, a 300 nm translation of the piezoelectric stage defocused the sample. A Fresnel zone plate was then created on the SLM to restore focus.

A Fresnel pattern is defined by its radius, $R_{f}$, and power, $p_{f}$ (see Equations 1 and 2 of the Supplementary Information) with focus control determined by changes in these parameters.^33^ While $R_{f}$ is an integer due to the discrete pixelated nature of the SLM, $p_{f}$ is a real number than can take any value. To quantify the focus control in our system, the evolution of image contrast for 40 nm AuNPs was plotted as a function of $p_{f}$. We compared these data with the evolution of image contrast as a function of z axis movement caused by a direct translation of the sample using the piezoelectric stage (Figure 3B). Specific SLM patterns are depicted in Figures S2 and S3 of the Supplementary Information. As our SLM is an 8‐bit digital device, ultimately we are limited to step changes in $p_{f}$ of 1/256, hence $\delta p_{f}\approx$ 0.004 (see Equations 1 and 2 of the Supplementary Information). With reference to the calibration curve in Figure 3A, this corresponds to a theoretical change in the focal plane position δz = 0.45 nm. For comparison, our piezoelectric control of z has a precision of δz = 100 nm. Focus control using the SLM has another benefit besides this improved precision; no mechanical part is required to move. Fast focus control is possible – limited by the refresh rate of the SLM display (30 Hz in our current setup, but other commercially available SLMs can achieve rates above 500 Hz), rather than relying on piezo‐control of focussing (here, ≈2.5Hz).

### 2.4 Background correction

In addition to direct focus control, we also considered the use of Fresnel patterns for fast on‐the‐fly background correction for iSCAT. Detection in iSCAT is often challenging due to the small fractional contrast associated with the interferometric scattering, and thus can be lost in background noise (typically ≤ 1%). To some extent this problem is alleviated by post‐processing, typically by median image division. However this is difficult to implement during image acquisition as: (i) it requires many images to be averaged (> 100), (ii) computing the median image for subsequent stack division is time consuming and negatively impacts frame rate, (iii) background correction by median division removes static objects that may be of interest from the image. Postprocessing Gaussian blurring has previously been used to provide a simple, means of background correction.^34^ Here we exploit the fast defocusing provided by Fresnel zone plates to provide background correction which is unencumbered by these typical drawbacks. Background correction by SLM fast defocusing does however represent a trade‐off and slower methods of excluding background by sample spatial or temporal displacement typically provide more efficient correction.

We again examined 40 nm AuNPs using the same particle detection conditions and preparation methods as used in Figure 2. A focused iSCAT image was collected and then a Fresnel pattern was applied to completely defocus the image, such that objects are no longer discernible while the pattern is displayed. Background correction is then simply achieved by processing in which the Fresnel pattern is consecutively applied and removed at a frequency equal to the frame rate of image acquisition of the camera detector. Alternating images, in and out of focus, are recorded by the camera, and the live focused image is continuously divided by the defocused image. An example of the corrected image this process would produce is given in Figure 3C. Since only two frames are required for this live background correction, we achieve a final frame rate of 30 Hz. Faster rates than this would be easily accessible with other pairings of CMOS detectors and SLM displays.

### 2.5 Orientation detection

In addition to directionless band‐pass filters, the SLM can be used to manipulate the Fourier space in an optical setup to select only a specific direction of the spatial frequency and cut all the other directions. Effectively, this results in an interference contrast reduction in the image in real space for all objects which have an orientation different to the one selected in the filter.

**FIGURE 4:**
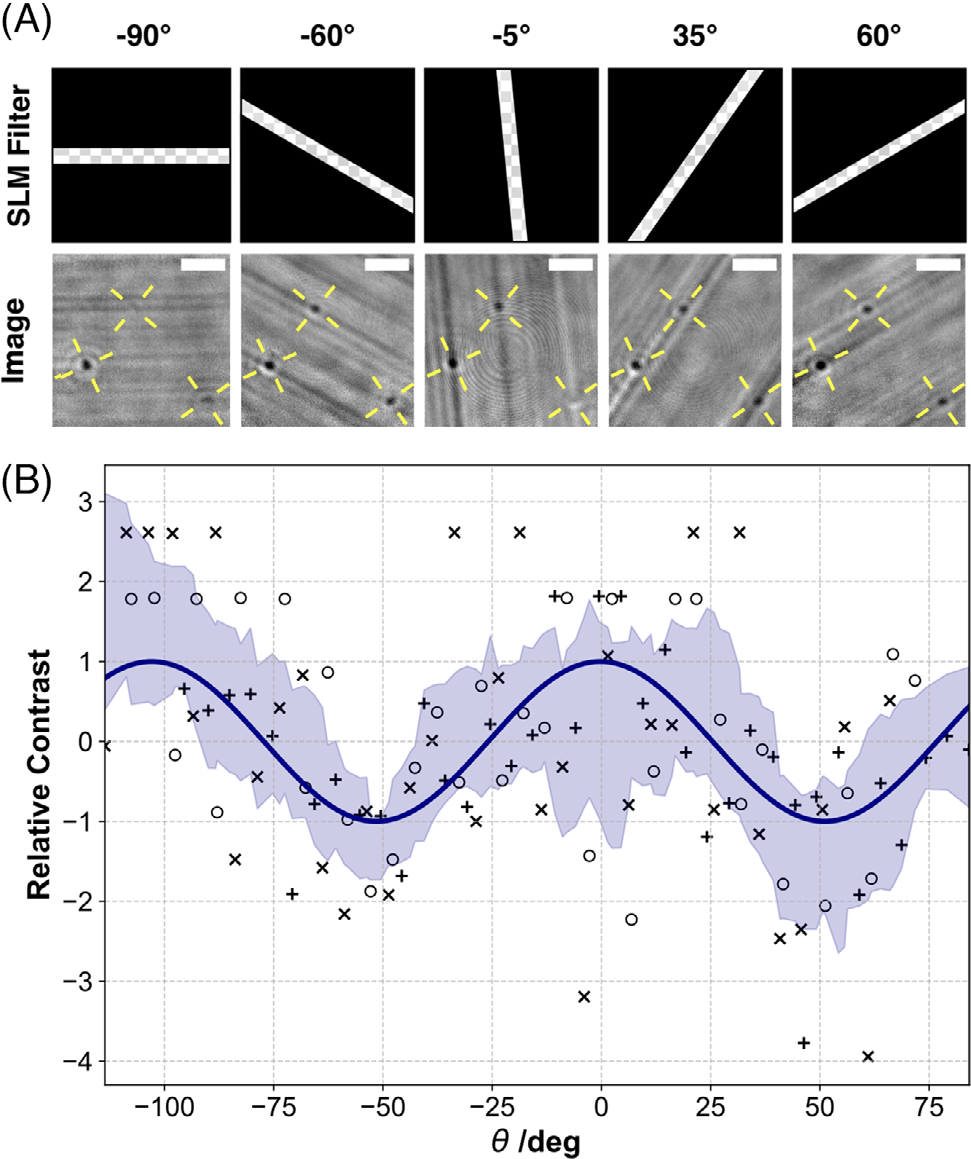
(A) Effect of linear filters set at different angles on visualising a sample of AuNRs. Illustration of the corresponding filters are shown on the top row. The yellow axes show the measured orientation of the corresponding objects. Scale bar is 2 μm. (B) Evolution of the contrast of AuNRs with the filter angle θ projected on the SLM. Each marker is a single data point, markers correspond to different AuNRs, area is the standard deviation CI, and the line is the sine fit of the individual points.

Gold nanorods (AuNRs) (length 40 nm, diameter 25 nm), chosen for their strong directional scattering due to their symmetry, were spin‐coated on a glass coverslip and imaged with the iSCAT. Because of their diffraction‐limited size, the AuNRs appear similar to spherical nanoparticles (Figure 4A) and it is not possible to tell their orientation directly from the image. A directional filter, consisting of a band of predetermined angle and thickness centred on the SLM display, was projected at varying angles. The image response to SLM band rotation is show in Figure 4B. The contrast of individual objects changed when the filter was rotated, eventually leading to individual particles completely disappearing from the image for certain orientations of the filter. Modulation of particle contrast upon filter rotation was not observed for spherical particles (Figure S4).

Measurement of contrast evolution for the AuNRs was executed by rotating the SLM‐projected band at intervals of 5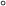 between –90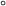 and +90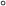. A total rotation of 180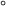 was selected because we assumed the AuNRs would behave with rotational symmetry of order 2 so all possible rotations can be described between 0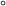 and 180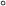. These data show diffraction limit particles, whose individual contrast varies with the rotation of the SLM filter. Different AuNRs reaching maxima at different rotations, corresponding to their (random) orientation on the surface. To determine the angular dependence of contrast, individual randomly oriented AuNRs were combined by aligning signal maxima – effectively ‘phase‐shifted’ all signals to match one another, then merged for an ensemble evolution of the signal and its period. Results are shown in Figure 4. The angular dependence of the relative contrast of the particles evolves in a sine wave shape with the orientation of the filter. A fit of the raw data of relative contrast using a simple sine function returns a period close to 90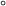. AuNRs have 2 main axes of line symmetry, perpendicular to each other, which would be expected to correspond to $90^{\circ }$ rotations of the directional filter. Our observations are consistent with signals modulating between these two contrast maxima every $∼90^{\circ }$, with an intermediate minima at $∼45^{\circ }$ to both axes.

## 3 CONCLUSIONS

In these experiments, we have sought to evaluate the advantage of PSF engineering for iSCAT microscopy. The direct dynamic access to the frequency domain of an image provided by SLMs offers many possibilities for interference contrast enhancement, background removal, and access to additional information, such as subdiffraction limited particle orientation. The use of iSCAT SLM provides speed, precision and versatile filtering without macroscopic perturbation of the optical system. Here, our use of high‐pass filtering showed a size‐independent optimal frequency as we chose to maintain sample consistency across the use of different filters. For nondiffraction limited objects, however, we expect dynamic control of spatial frequency cutoff would become increasingly important. We used linear filters to determine the orientation of diffraction limited AuNRs; however, the temporal modulation in intensity provided by this approach might also provide a future route to optical heterodyne detection of iSCAT signals.

Beyond the applications covered in this work, we foresee numerous possibilities for implementations relevant to interferometric microscopy, exploiting the large diversity of PSF engineering available: for instance, a displayed diffraction grating pattern can produce image duplicates to process features in parallel^35^; a VanderLugt correlator might be applied to detect specific features in the sample^36^; label‐free 3D particle tracking is also becoming an area of interest in iSCAT research^37^; and we see the SLM as having potential for adaptive wavefront control to enable future 3D applications.

## 4 METHODS

### 4.1 Materials

Gold nanoparticles (AuNPs) of size ranging from 5 to 40 nm, gold nanorods (AuNRs) of length 40 nm, diameter 25 nm, and solvents used in this work were purchased from Sigma‐Aldrich (now Merck, Darmstadt, GE).

### 4.2 Sample preparation

Borosilicate glass coverslips (24×60 mm, #1 thickness, Menzel Gläser) were sonicated for 15 min in Decon 90 (10% v/v, Fisher Scientific, Hampton, NH, USA) and washed 8× in purified water (Millipore Direct‐Q UV3, Merck). Coverslips were sonicated for a further 15 min in water, washed 8× in water, and stored in isopropyl alcohol.

Coverslips were dried under a stream of nitrogen and treated with an oxygen plasma for 5 min (Diener Electronic, 90 W, 0.5 bar oxygen flow). Following cleaning, AuNPs were sonicated for 2 min to encourage breakup of particle aggregates. Coverslips were then spin coated twice at 4000 rpm for 30s (...) with 50 μL of AuNPs suspension without further dilution, for a total of 100 μL of AuNPs deposited on the surface of each coverslip.

To image the sample, a silicon spacer (Coverwell, Grace Bio‐Lab, Bend, OR USA) was installed on top of the coverslip and filled with water. A second coverslip (18 mm diameter, Chongqing New World Trading Co.) was cleaned using the procedure described above. The top of the observation chamber was then sealed with this coverslip.

### 4.3 Image acquisition and analysis

Data acquisition was controlled using LabVIEW (National Instruments, Austin TX USA). For the data presented here, 300 frames were recorded at 150 Hz. Laser power was set to the maximum available (80 mW). The exposure time of the camera was then set automatically by our control software to ensure the maximum pixel value detected was 85%–90% of the pixel full well capacity of the detector to prevent saturation and maximise the accuracy of the contrast measurement.

Image analysis was performed using Python scripts developed in‐house. All measurement were preceded by image normalisation via division of each frame by the median‐averaged projection. Particles were then located using the Python module TrackPy.^31^ Where required, a linear profile was plotted across the particle and fitted using a sinc function. The amplitude of the fitted sinc determines the measured intensity of particle signal ($I_{s}$) and background ($I_{b}$) respectively. Particle contrast was calculated as $C=\left(I_{s}-I_{b}\right)/I_{b}$.

## Supporting information

Supporting Information
