# Supplementary material for: Spatial light modulation for interferometric scattering microscopy
Source: J Microsc. 2024 Aug 26;297(1):88–95. doi: 10.1111/jmi.13347 (PMC11629933; doi:10.1111/jmi.13347)
Supplement: Supplementary file 1 — Supporting Information [file JMI-297-88-s001.pdf]

Point spread function engineering for interferometric scattering microscopy

Vivien Walter, Christopher Parperis, Mark Ian Wallace.

Supplementary Note 1: Highpass filter design

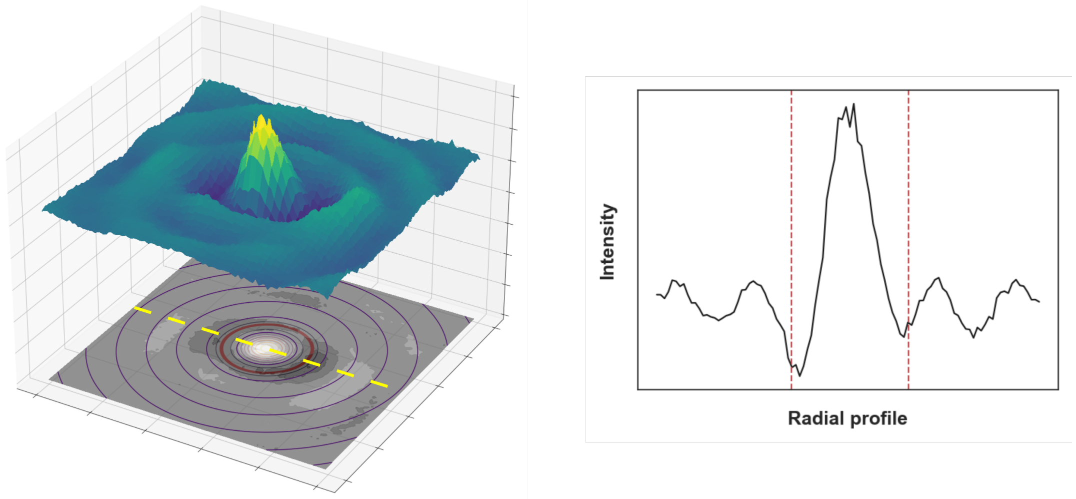

Fig. S1. (Left) 3D representation of the intensity profile of a gold nanoparticle as observed in iSCAT. The 2D image of the particle is show projected at the bottom along with the contour of the sinc fit of the profile (blue lines). The red ring represents the period of the limit of the optimal highpass filter found experimentally (997 m^{-1}). (Right) 2D intensity profile taken along the yellow dashed line of the 3D projection. The period of the filter is shown as red dashed lines.

Supplementary Note 2: Fresnel lens pattern design

A. Construction of the pattern. The shape of the Fresnel zone plate on the SLM model used in this work is modulated using two parameters:

- The radius of the lens R_f, given in pixel units.
- The "power" of the lens p_f, corresponding to the steepness of the lens at a given position.

Based on these two parameters, the Fresnel lens pattern is then calculated using the equation (1), describing the profile of the pattern at each position $(x;y)$ away from the center of the lens.

$$A_{(x,y)} = \frac{p_f}{2}\left(2\left(\left(\frac{x}{R_f}\right)^2 + \left(\frac{y}{R_f}\right)^2\right) - 1\right) \quad \textbf{(1)}$$

Several linear profiles drawn by this equation for different couples of (R_f;p_f) are shown in Figure S2. To fit the 2D profile on a 8-bit LCD display, the pattern is cut into zones of intensity increasing only from 0 to 1, using equation (2).

$$I_{(x,y)} = A_{(x,y)} - \lfloor A_{(x,y)} \rfloor \quad \textbf{(2)}$$

The resulting intensity $I_{(x,y)}$ is then multiplied by 255 and rounded to the nearest integer to obtain the pixel values to be displayed on the SLM. The linear profiles drawn by this equation for the different couples of (R_f;p_f) are shown in Figure S2(b).

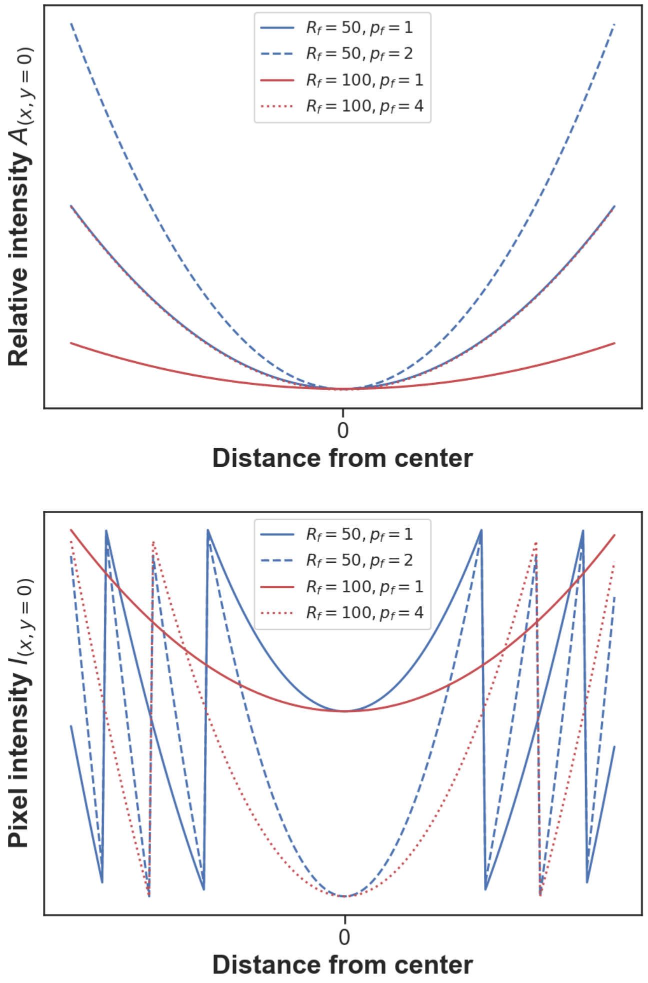

Fig. S2. Evolution of the intensity profiles (a) $A_{(x,y)}$ and (b) $I_{(x,y)}$ used to generate the Fresnel lens patterns on the SLM, for different values of the Fresnel lens radius R_f and power p_f.

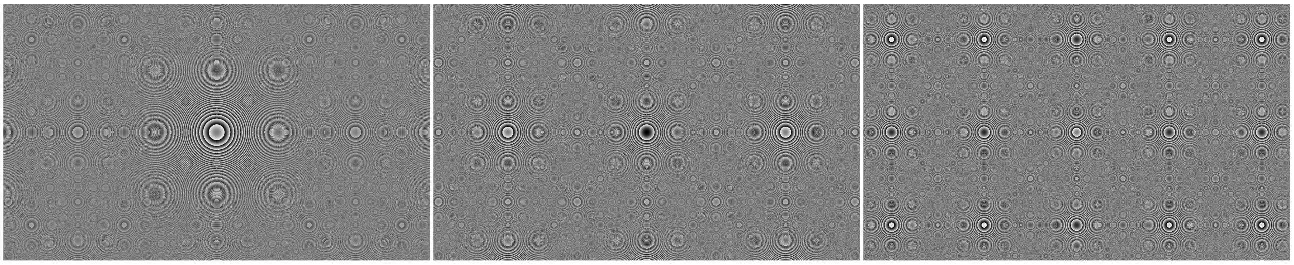

(a) R_f = 50, p_f = 1.0 **(b)** R_f = 50, p_f = 2.0 **(c)** R_f = 50, p_f = 3.0

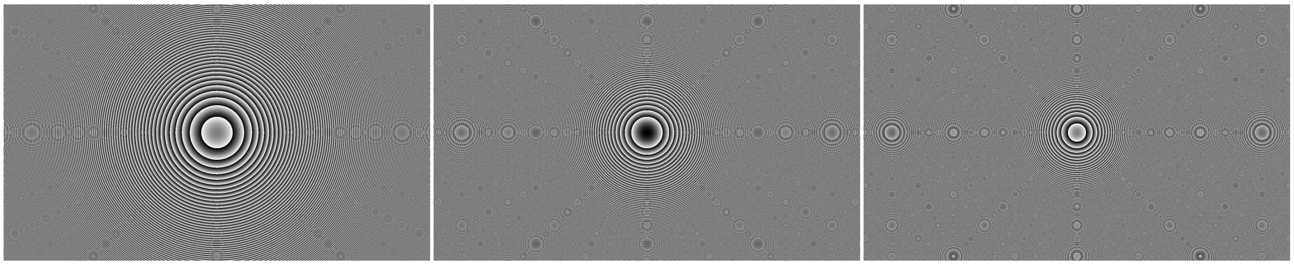

(d) R_f = 100, p_f = 1.0 **(e)** R_f = 100, p_f = 2.0 **(f)** R_f = 100, p_f = 3.0

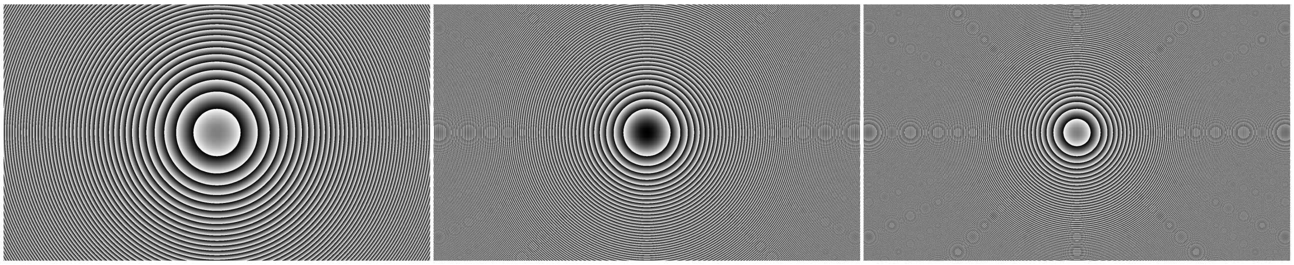

(g) R_f = 150, p_f = 1.0 **(h)** R_f = 150, p_f = 2.0 **(i)** R_f = 150, p_f = 3.0

Fig. S3. Fresnel lens pattern generated by the SLM control software for different values of the Fresnel lens radius R_f and power p_f, using the equation (3) of the main text.

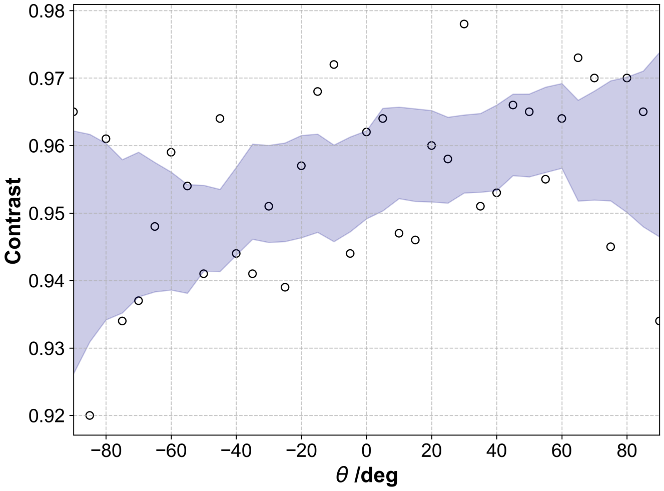

Fig. S4. Evolution of the contrast with angle for AuNP. Control experiments show no angle dependence in contrast for linear SLM filtering of 40nm AuNP.
